# The Emerging Roles of Ferroptosis and NETosis in Pregnancy Complications: Insights into Preeclampsia and Gestational Diabetes Mellitus

**DOI:** 10.3390/cimb47090685

**Published:** 2025-08-25

**Authors:** Vasiliki Katsi, Angeliki Alifragki, Konstantinos Fragkiadakis, Nikolaos Kopidakis, Eleutherios Kallergis, Evangelos Zacharis, Emmanouil Kampanieris, Emmanouil Simantirakis, Konstantinos Tsioufis, Maria Marketou

**Affiliations:** 1 1st Department of Cardiology, School of Medicine, National and Kapodistrian University of Athens, Hippokration General Hospital, 115 27 Athens, Greece; vkkatsi@yahoo.gr (V.K.);; 2 School of Medicine, University of Crete, 700 13 Iraklion, Greece

**Keywords:** ferroptosis, NETosis, pregnancy, complications, preeclampsia, gestational diabetes mellitus

## Abstract

Gestational complications, such as preeclampsia and gestational diabetes mellitus (GDM), pose significant risks to maternal and fetal health and increase long-term cardiovascular disease risk in offspring. This review aims to synthesize current knowledge on the roles of ferroptosis and neutrophil extracellular trap formation (NETosis)—two regulated cell death pathways—in these pregnancy-related conditions. We performed a comprehensive analysis of preclinical and clinical studies that investigate the involvement of dysregulated iron metabolism, oxidative stress, inflammation, and endothelial dysfunction mediated by ferroptosis and NETosis in gestational pathologies. Evidence indicates that disturbances in maternal iron homeostasis and enhanced formation of lipid peroxides and NETs contribute to placental dysfunction and systemic inflammation, exacerbating disease severity. Therapeutic strategies targeting these pathways are emerging but require further validation. Our review also identifies key gaps in mechanistic understanding, biomarker development, and translational research needs. We conclude that modulation of ferroptosis and NETosis offers promising avenues for improving diagnosis and treatment of pregnancy complications, though carefully designed clinical studies are essential to confirm their clinical utility and safety.

## 1. Introduction

**Figure 1:**
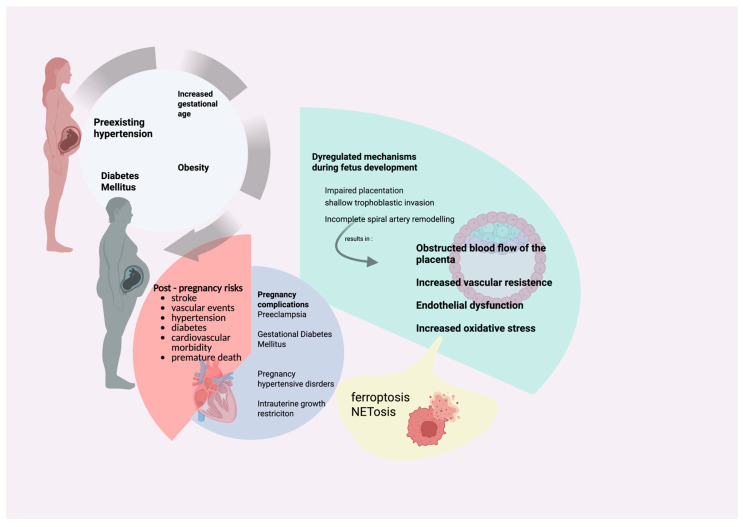
Overview of common risk factors and mechanisms of pregnancy complications. Common described risk factors leading to pregnancy complications is preexisting hypertension, increased gestational age, diabetes mellitus and obesity among others. Those individuals have increased risk for pregnancy complications like preeclampsia or gestational diabetes mellitus but also post-pregnancy morbidity with higher risk for stroke or other vascular events. On the right side of the illustration are presented few of suggestive dysregulated mechanisms while common pathways in several cases are found to be ferroptosis and NETosis. (This illustration was created using the icon library of https://app.biorender.com/).

Cardiovascular diseases occur in approximately 1–4% of pregnancies and represent a major cause of maternal morbidity and mortality worldwide [1]. As the maternal age continues to rise mainly in high-income countries, both the incidence and complexity of these conditions are increasing-contributing not only to adverse outcomes for mothers and their offspring, but also to a growing economic burden due to prolonged hospitalizations and long-term care needs [2]. Importantly women affected by gestational complications of increased cardiovascular risk during pregnancy remain at heightened risk for stroke, vascular disease, and premature mortality for years after delivery, underscoring the need for long-term surveillance and prevention strategies [3] (Figure 1).

Among pregnancy-related conditions, preeclampsia (PE)-new-onset hypertension and proteinuria or end-organ dysfunction after 20 weeks of gestation [4] and gestational diabetes mellitus (GDM)—defined as glucose intolerance and insulin resistance first recognized during pregnancy [5]—are strongly associated with increased long-term cardiovascular risk that frequently co-exist and share several pathophysiological mechanisms, including placental dysfunction, immune dysregulation and endothelial injury [6]. Furthermore, in some cases, gestational diabetes may predispose to preeclampsia through persistent hyperglycemia and resulting endothelial damage, suggesting a sequential and mechanistic link between the two [7].

Recent insights from molecular and immunological research have identified two emerging processes—ferroptosis, a form of regulated, iron-dependent cell death characterized by lipid peroxidation [8], and NETosis, the formation of neutrophil extracellular traps (NETs) [9]—as novel contributors to cardiovascular pathology [10,11]. While their roles are increasingly recognized in non-pregnancy-related cardiovascular diseases, their impact during pregnancy remains poorly defined. Notably, excessive neutrophil activation and immune cell infiltration have been implicated in both GDM and PE, supporting an inflammatory axis that may drive disease progression [12]. Excessive ferroptosis is also linked to heightened systemic inflammation and impaired angiogenesis, further exacerbating cardiovascular risk in affected pregnancies highlighting the direct role of this type of cell death with significant cardiovascular risk [13].

Given the unique immunological and physiological environment of gestation, elucidating the contributions of ferroptosis and NETosis to preeclampsia and gestational diabetes mellitus could uncover novel therapeutic targets and improve maternal and neonatal outcomes.

In this review, we summarize current knowledge on the participation of ferroptosis and NETosis in the development of GDM and PE; we discuss their clinical implications, potential as therapeutic targets, and propose directions for future research that bridge basic science and clinical care.

## 2. Methods

This narrative review aimed to explore the mechanistic roles of ferroptosis and NETosis in the pathophysiology of pregnancy complications, with a primary focus on preeclampsia and gestational diabetes mellitus (GDM). Relevant literature was identified through a comprehensive search of PubMed, Google Scholar, and Scopus, covering publications from 2012 to August 2025. The following keywords were used in various combinations: “*ferroptosis*,” “*NETosis*,” “*gestational diabetes mellitus*,” “*preeclampsia*,” “*hypertensive disorders*,” and “*gestational pathology.*” Studies were included if they focused on the molecular or cellular mechanisms of ferroptosis and/or NETosis in the context of preeclampsia and GDM. Preference was given to recent and peer-reviewed original research articles and reviews. Articles not directly addressing the role of these pathways in pregnancy complications were excluded. Selected studies were analyzed thematically to synthesize current knowledge and identify emerging patterns or gaps in the literature.

## 3. Foundations and Failures of Uteroplacental Circulation

Pregnancy involves complex anatomical and physiological adaptations in both the mother and the fetus, extending from early gestation through the postpartum period [6]. A central feature of this process is the placenta, which plays a pivotal role in fetal growth and survival by regulating key metabolic processes, including iron homeostasis, glucose uptake, and insulin signaling in trophoblast cells [14,15,16]. Proper placental development—especially its vascular components—is essential for establishing the low-resistance uteroplacental circulation that supports continuous fetal nourishment [17].

At the foundation of this vascular transformation is the two-phase process of trophoblastic invasion, classically referred to as the ‘first’ and ‘second waves’ [18]. The first wave involves early migration of extravillous trophoblasts into the decidua and superficial myometrium under hypoxic and hypoglycemic conditions [18]. This is followed by the second wave, occurring in the late first and early second trimesters, during which deeper trophoblastic infiltration into the myometrial segments of the spiral arteries enables structural remodeling [19]. Successful transformation results in fully dilated, low-resistance vessels capable of sustaining fetoplacental perfusion.

This remodeling process includes an initial phase of transient vascular occlusion by cellular debris and thrombi, which are later cleared to restore luminal patency [17]. When this sequence is impaired—due to inadequate trophoblastic invasion or incomplete spiral artery remodeling—a Persistent luminal obstruction can maintain a hypoxic intrauterine environment, predisposing the maternal–fetal interface to hypoxia–reperfusion injury. This phenomenon mirrors the ischemia–reperfusion injury observed in myocardial infarction, wherein sudden reoxygenation of previously ischemic tissue provokes inflammatory and oxidative responses [17]. Recanalization of spiral arteries shares mechanistic parallels with this process, as the abrupt exposure of developing fetal tissues to maternal-derived oxygen and metabolites can likewise trigger cellular stress and excess inflammatory responses [17].

## 4. Ferroptosis in Pregnancy

**Figure 2:**
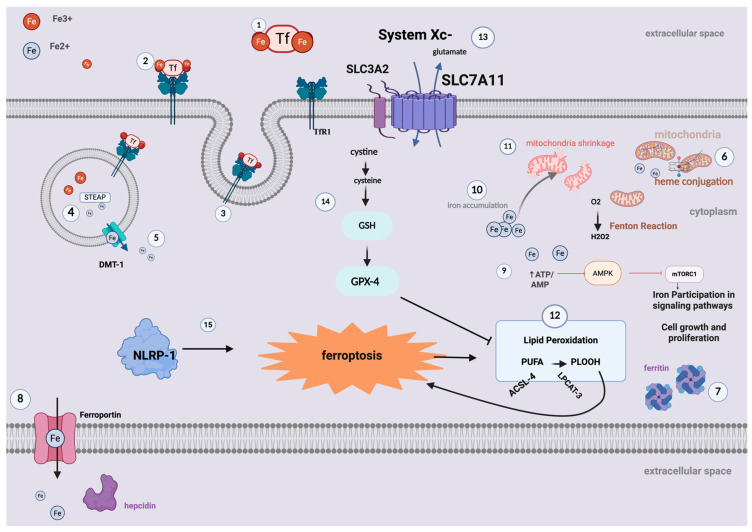
Molecular pathways of iron and shared pathways with ferroptosis. In this schematic illustration, we present iron absorption and molecular regulatory pathways that enhance or inhibit ferroptosis. Iron binds to circulating transferrin in its ferric state (1). Transferrin subsequently binds to transferrin receptors located on the cell membrane (2). The transferrin–transferrin receptor complex (mainly TfR1) is then endocytosed (3). In acidified endosomes within the cytoplasm, ferric iron (Fe^3+^) is converted to the ferrous state (Fe^2+^) (4) and is released into the cytoplasm via the divalent metal transporter 1 (DMT-1) (5). Iron participates in several cellular metabolic pathways, including heme synthesis in the mitochondria (6). It can also be stored in ferritin to prevent accumulation or degradation that may trigger oxidative stress (7). Iron can exit the cytoplasm via Ferroportin, which is located on the cell membrane (8). Hepcidin binds to Ferroportin with high affinity and specificity, thereby inhibiting iron export from the cell. Iron also directly participates in signaling pathways such as AMPK/mTOR (9). Ferroptosis is driven by iron accumulation (10) and leads to organelle damage (11), lipid peroxidation (12), and ultimately cell death. Lipid peroxidation is mediated by ACSL-4 and LPCAT-3, leading to the production and accumulation of phospholipid hydroperoxides (PLOOH) through the breakdown of bonds in polyunsaturated fatty acids (PUFAs), thereby enhancing ferroptosis (12). System Xc^−^, composed of two subunits (SLC3A2 and SLC7A11), acts as a glutamate/cystine antiporter (13). Once inside the cell, cystine is converted to glutathione (GSH), and glutathione peroxidase-4 (GPX-4) inhibits lipid peroxidation (14) and, ultimately, ferroptosis. NLRP-1 activation further enhances ferroptosis (15). (This illustration was created using the icon library of https://app.biorender.com/).

Ferroptosis was first described by Dixon et al. in 2012 [8] as a distinct form of regulated cell death characterized by intracellular iron accumulation and excessive oxidative stress, particularly through the overproduction of reactive oxygen species (ROS). These factors contribute to lipid peroxidation, disruption of cellular membranes, and organelle damage, notably mitochondrial shrinkage and loss of structural integrity. Lipid peroxidation primarily affects polyunsaturated fatty acids (PUFAs), key constituents of cellular membranes, which are susceptible to oxidative cleavage via the Fenton reaction. This process leads to the formation of lipid hydroperoxides that cannot be efficiently metabolized, ultimately triggering ferroptotic cell death [8] (Figure 2).

### 4.1. Iron Metabolism: Pathways and Key Regulatory Molecules

Iron is an essential trace element involved in numerous physiological processes. It is widely available through dietary sources and is commonly administered as a supplement in various medical conditions and during pregnancy [20,21]. In the human body, iron primarily exists in the ferric form (Fe^3+^) and must be reduced to its ferrous state (Fe^2+^) to participate in critical biochemical reactions (Figure 2) [20]. When circulating in plasma, iron in Fe^3+^ state can be bind to transferrin-a plasma protein that carries 2 atoms of iron per molecule and delivers iron to distal tissues for use [22]. Transferrin binds to transferrin receptors–mainly TfR1 expressed in every cell-and the complex Tf-TfR1 is endocytosed in acidified endosomes where under the influence of ferrireductase STEAP, iron converts to Fe^2+^ leaving transferrin [23]. From endosomes, Fe^2+^ is transported through DMT-1 (Divalent Metal Transporter 1), which is located on the surface of endosomes in the cytoplasm of duodenal enterocytes and is primarily responsible for iron release inside the cell [24]. Fe^3+^ can also be reduced to the Fe^2+^ state by duodenal cytochrome b (Dcytb), a ferric reductase located on the apical membrane of duodenal enterocytes [25]. The resulting Fe^2+^ is subsequently transported into the cell by divalent metal transporter 1 (DMT1), which exclusively carries iron in its ferrous form from the apical membrane into the cytosol. Based on cell needs, iron follows relative pathways. It can be directly transported to mitochondrial membrane, where it conjugates with heme, contributing to heme biosynthesis [26] or it can be stored inside the cell bind with ferritin-large intracellular protein capable to storage large quantities of iron [26]. Excess intracellular iron may be exported from cell to the plasma via Ferroportin-the only known mammalian iron exporter-highly expressed in proximal duodenal cells [27].

Iron is tightly regulated by other molecules and peptides found inside the cell or circulated in plasma. Hepcidin, a well-characterized peptide hormone synthesized in the liver, plays a central role by binding to Ferroportin with high affinity and specificity [28]. In this context, hepcidin-mediated inhibition of Ferroportin leads to intracellular iron retention, potentially enhancing iron’s biological effects and therefore potentially enhances ferroptosis [28]. Interestingly, hepcidin is also utilized by bacteria and functions in inflammation as well as in triggering erythropoiesis [29]. Furthermore, intracellular iron homeostasis is governed by iron regulatory proteins (IRPs), which modulate gene expression in response to fluctuating iron levels [26]. Under iron-deficient conditions, IRPs bind to iron-responsive elements (IREs) within the untranslated regions of target mRNAs, stabilizing transcripts such as those encoding the transferrin receptor to enhance iron uptake, while concurrently inhibiting the translation of ferritin to limit iron sequestration [26]. Conversely, in iron-replete states, IRP binding diminishes, leading to reduced transferrin receptor synthesis and increased ferritin expression, thereby promoting iron storage. This post-transcriptional regulatory network ensures precise control of iron metabolism (Table 1).

**Table 1:** Summary of key molecular markers involved in ferroptosis and NETosis pathways, their roles, and associations with preeclampsia (PE) and gestational diabetes mellitus (GDM).

Pathway	Key Molecular Marker	Molecular Name	Mechanistic Role	Association with PE	Association with GDM	Reference
Ferroptosis	NDRG1	N-myc downstream-regulated gene 1	Promotes tumor survival by inhibiting ferroptotic cell death	Upregulation in PE	NR	He et al. [30]
	P4HA1	Prolyl 4-hydroxylase subunit alpha 1	Mevalonate pathway Activates HMGCS1 Promoting proliferation and metastasis Protects cell from erastin induced ferroptosis	Upregulation in PE	NR	He et al. [30]
	LDHA	Lactate dehydrogenase A	Drives glycolysis and lactate production-resistance to ferroptosis	Upregulation in PE	NR	He et al. [30]
	IDO-1	Indoleamine 2,3-dioxygenase 1	Kynurenine pathway Catalyzes tryptophan degradation to kynurenine Suppresses ferroptosis indirectly through scavenging ROS and activating NRF2.	Downregulation in PE	NR	He et al. [30]
			Promotes vasodilation through degradation of tryptophan to kynurenine	Downregulation in PE	NR	He et al. [30]
	ALB	Albumin	Native albumin mediates antioxidant effects Oxidized albumin promotes ferroptosis by increasing intracellular iron	Increased expression in PE	NR	Wu et al. [31]
	CDKN2A	Cyclin-dependent kinase inhibitor 2A	Activates JAK2/STAT3 signaling pathway and upregulates anti-ferroptotic proteins	Increased expression in PE	NR	Wu et al. [31]
	TXNRD1	Thioredoxin reductase-1	Cytosolic selenoenzyme with antioxidant features Prevents accumulation of lipid hydroperoxides	Increased expression in PE	NR	Wu et al. [31]
	CAV1	Caveolin-1	Membrane scaffolding enzyme regulates lipid peroxidation & expression of GPX-4 and SLC7A11 Overexpression inhibits ferroptosis by reducing ROS	Increased expression in PE	NR	Wu et al. [31]
	SRXN1	Sulfiredoxin-1	Activates HO-1 Promotes ferroptosis	Downregulation in PE	NR	Wei et al. [32]
	NOX 1/2	NADPH Oxidase 1/2	Production of superoxide Positive regulator of ferroptosis	Increased expression in PE	NR	Wu et al. [31]
	NOX4	NADPH Oxidase-4	Production of hydrogen peroxide and superoxide and promotes ferroptosis	Increased expression in PE	NR	Wu et al. [31]
	ACSL4	Acyl-CoA synthetase long-chain family member 4.	Ligation of PUFAs	Increased expression in PE	NR	Ortega et al. [33]
	GPX4	Glutathione peroxidase 4	Central defender against ferroptosis Reduction of PLOOHs to non-toxic lipid alcohols using GSH as cofactor	Increased expression in PE	Increased levels in GDM driven by upregulation of SIRT3	Ortega et al. [33]
	SLC7A11	Solute carrier family 7-member 11	Light chain subunit of system Xc- Cystine/glutamate antiporter Regulates levels of cystine imported inside the cell	Increased expression in PE	Increased levels in GDM driven by upregulation of SIRT3	Ortega et al. [33]
	TfRC	Transferrin Receptor	Cellular iron uptake after binding with circulated transferrin	Increased expression in PE	NR	Ortega et al. [33]
	SIRT 6	Sirtuin 6	Upregulates NRF-2/HO-1 axis leading to increased expression of antioxidant genes	Elevated levels in PE related with stimulated pathway of NRF-2/HO-1	NR	Qi et al. [34]
	FPN	Ferroportin	Iron exporter of the cellular membrane Exports Fe^2+^ from the cell decreasing iron accumulation	Reduced expression in pregnancies complicated with PE	NR	Ng. et al. [35]
	IRS 2 /IRS 3	Insulin-receptor substrate 2/3	Mediates downstream of PI3K/AKT Modulates glucose metabolism via AKT-mediated NRF2-activation Promotes synthesis of GSH and reduces oxidative stress Inhibit ferroptosis	NR	Serine residuals phosphorylation inhibits GLUT incorporation in cell membrane sustaining hyperglycemia resulting in GDM	Metz and Houghtonet al. [36]
	mTOR	Mechanistic target of rapamycin	Upregulation of SREBP-1, enhances lipid biosynthesis and maintains redox homeostasis Negative regulator of ferroptosis	Hyperactivation of mTOR pathway promotes insulin resistance and indirect hyperglycemia predisposing in GDM and PE		Saxton et al. [37]
	LPCAT3	Lys phosphatidylcholine acyltransferase 3	Incorporates PUFAs into membrane phospholipids promoting ferroptosis	NR	Increased lipid peroxidation impairs trophoblast invasion and enhances ferroptosis that precipitates in GDM	Du et al. [38]
*NETosis*	ASK-1 inflammasome	Apoptosis signal-regulating kinase 1	Promotes apoptosis and inflammation	Increased oxidative stress and inflammation activate ASK-1, which can amplify trophoblast apoptosis and placental injury, contributing to PE and GDM		NaveenKumar et al. [39]
	L-Selectin		Leukocyte adhesion molecule participates in neutrophils’ recruitment	Amplify inflammation and oxidative stress predispose to PE and GDM		
	ICAM-1	Intercellular adhesion molecule 1				Luppi et al. [40]
	IL-8	Interleukin-8	Neutrophil chemoattractant protein induces NETs formation			Leik et al. [41]
	NE	Neutrophil elastase	Translocates to cell nucleus and it cleaves histones and further promotes chromatin relaxation.	Elevated levels detected in PE and associations with level-dependent disease severity		Kenny et al. [42]
	MPO	Myeloperoxidase	Facilitates both NE nuclear translocation and chromatin decondensation			Kenny et al. [42]
	HMGB-1	High-mobility group box 1	Promotes inflammation and oxidative stress resulting in trophoblast injury	Enhanced oxidative stress in placental interface of pregnancies complicated with PE	Increased levels in maternal serum and predisposes to insulin resistance	Dong et al. [43]

Abbreviations in the table: PE: Preeclampsia, GDM: Gestational Diabetes Mellitus, NR: Not reported, HMGCS1: 3-hydroxy-3-methylglutaryl-CoA synthase 1, NRF2: nuclear factor erythroid 2-related factor 2, JAK2/STAT3: Janus kinase 2/STAT3: signal transducer & activator of transcription 3, HO-1: heme oxygenase 1, PUFAs: Polyunsaturated fatty acids, PLOOHs: phospholipid hydroperoxides, GSH: glutathione, SIRT3: Sirtuin 3, PI3K: phosphoinositide 3-kinase, AKT: protein kinase B, SREBP-1: sterol regulatory element-binding protein 1, NETs: neutrophil extracellular traps.

### 4.2. Placental Iron Transport: Links Between Ferroptosis, Iron Imbalance, and Placental Dysfunction

Iron plays a vital role in fetal growth and neurodevelopment, supporting numerous intracellular processes in placental and fetal tissues [44]. During pregnancy, maintaining iron homeostasis is critical: both iron deficiency and iron overload have been linked to adverse outcomes, including preterm birth and low birth weight, suggesting a U-shaped relationship between maternal iron status and fetal health [45]. While iron deficiency is widely recognized and routinely treated, iron overload remains under-recognized and insufficiently investigated, despite growing evidence implicating it in gestational pathologies [45,46].

At the placental interface, iron transfer to the fetus is mediated by mechanisms analogous to systemic iron handling. The predominant pathway involves maternal transferrin binding to transferrin receptors on the syncitiotrophoblast membrane, followed by receptor-mediated endocytosis. Within endosomes, iron is released and transported into the cytoplasm in its ferrous (Fe^2+^) form, via divalent metal transporter 1 (DMT1), where it is either stored in ferritin or exported into the fetal circulation via Ferroportin [47].

This mechanistic overlap has heightened interest in ferroptosis and iron-regulatory molecular pathways as potential drivers of placental dysfunction and adverse pregnancy outcome [46]. Disruption in these tightly regulated iron pathways may contribute to oxidative stress, cellular injury, and impaired placental function—hallmarks of conditions such as preeclampsia and fetal growth restriction.

### 4.3. Maternal Adaptations to Iron Demands During Pregnancy

Pregnancy triggers tightly regulated adaptations in iron homeostasis to support fetal development while preserving maternal reserves [48]. One pivotal mechanism is the downregulation of hepcidin, a peptide hormone that binds to and degrades Ferroportin—the primary iron exporter—thereby enhancing iron transfer across trophoblast cells to the fetus [49,50]. In parallel, transcriptional adaptations include the upregulation of *transferrin receptor 1* (*TfR1*), particularly under maternal iron-deficient conditions, facilitating increased iron uptake during early gestation [51]. As placental development progresses, iron is either stored in placental ferritin or transferred to the fetus via transferrin-mediated pathways, resembling non-pregnant iron trafficking [52]. Additional transporters, such as ZIP8, also play critical roles in early fetal iron acquisition. Experimental ZIP8 knockdown in mice significantly reduces fetal iron uptake, suggesting its involvement in ferroptosis-associated pregnancy complications [53]. These regulatory networks intersect with ferroptosis, a form of iron-dependent cell death marked by ROS accumulation and lipid peroxidation. Reduced glutathione (GSH) and NADPH levels sensitize cells to ferroptosis, whereas elevated GPX4 mitigates oxidative damage and supports cell survival [54].

### 4.4. Cellular Defense Mechanisms Against Ferroptosis

Cells employ multiple defense mechanisms to prevent ferroptosis, a form of regulated cell death driven by iron-dependent oxidative damage. Central to this defense is glutathione peroxidase 4 (GPX4), which neutralizes lipid hydroperoxides using glutathione (GSH) as a cofactor [55]. GSH is synthesized from cysteine, which is derived from extracellular cystine imported via the system Xc^−^ antiporter, composed of SLC7A11 and SLC3A2. This antiporter exchanges intracellular glutamate for extracellular cystine, which is then reduced intracellularly to cysteine. Loss of GPX4 activity or GSH depletion sensitizes cells to ferroptosis, highlighting the essential role of system Xc^−^ in cellular redox homeostasis [55]. In parallel, iron export via Ferroportin, the only known mammalian iron efflux transporter, prevents intracellular iron accumulation and limits Fenton chemistry-mediated lipid peroxidation as mentioned above [49,50]. Lipid metabolism also influences ferroptosis susceptibility. Enzymes such as ACSL and LPCAT promote the incorporation of polyunsaturated fatty acids into membrane phospholipids, making them prone to peroxidation [56]. Thus, ferroptosis is tightly regulated by antioxidant defenses, iron handling, and lipid remodeling pathways.

### 4.5. Ferroptosis and Intracellular Pathways in Pregnancy Complications

Ferroptosis has emerged as a key contributor to pregnancy-related complications through its link to oxidative stress and dysregulated placental metabolism. Multiple studies have identified reduced levels of GPX4, superoxide dismutase (SOD), glutathione, and elevated levels of malondialdehyde (MDA), ACSL4, and NLRP1, implicating ferroptotic and inflammatory pathways in placental pathology [57]. During early pregnancy, increased mitochondrial content in trophoblast and placental cells enhances oxidative stress, contributing to sustained metabolic strain [58]. While this mitochondrial expansion is physiologically necessary, it demands a proportional increase in antioxidant capacity. If this adaptive response is overwhelmed or inhibited, ferroptosis may be triggered, contributing to adverse outcomes such as preeclampsia and intrauterine growth restriction (IUGR) [58]. Emerging research has also highlighted a link between cholesterol metabolism and oxidative injury. Ki Mo Lee et al. [59] reported that maternal cholesterol levels rise during pregnancy, supporting fetal development and generating oxysterols—oxidized cholesterol derivatives that influence lipid metabolism and cell survival. In trophoblasts, exposure to 25-hydroxycholesterol (25HC) induced abnormal placentation, metabolic disruption, and morphological changes consistent with oxidative injury and ferroptosis [59]. The role of the inflammasome, particularly NLRP1, has further clarified the intersection between ferroptosis and inflammation. In a study by Meihe et al. [60], placental tissues exposed to oxidative stress exhibited elevated expression of ferroptosis markers (e.g., TfR1, ACSL4) and inflammasome components. Silencing of NLRP1 restored levels of GSH and GPX4 and suppressed expression of oxidative stress markers. In contrast, NLRP1 activation exacerbated ferroptosis-related molecular changes. The study also implicated NLRP3, IL-1β, and caspase-1 in the ferroptotic response, establishing a direct link between inflammasome activation and ferroptosis in placental dysfunction [60,61]. These findings suggest that impaired antioxidant defenses, cholesterol-derived oxidative stress, and inflammasome signaling converge to amplify ferroptosis, offering mechanistic insights into the pathogenesis of pregnancy complications.

Having outlined the role of placental development in the setting of endothelial dysfunction and oxidative stress—key drivers of gestational pathology—we now turn to a focused examination of ferroptosis-related mechanisms in preeclampsia and gestational diabetes mellitus, which will be addressed independently in the sections that follow.

## 5. Preeclampsia Pathogenesis and Ferroptosis

Preeclampsia is a well-characterized pregnancy complication associated with significant long-term cardiovascular risk [62]. Clinically, it is categorized by gestational age at onset: early-onset preeclampsia occurs before 34 weeks, while late-onset presents thereafter. These subtypes differ not only in timing but also in underlying pathophysiology [63]. Early-onset preeclampsia is often linked to impaired placentation and defective spiral artery remodeling, whereas late-onset disease is more commonly observed and may reflect subtler maternal or placental dysfunction [63]. Several conditions—including abnormal implantation, hydatidiform mole, placentomegaly, fetal hydrops, and antiphospholipid syndrome—may precipitate preeclampsia, even in the absence of pre-existing maternal cardiovascular disease, which is frequently unremarkable in affected patients [63]. The predominance of late-onset cases in clinical practice may reflect the nonspecific or subclinical nature of early symptoms, leading to under recognition of earlier-onset disease despite ongoing pathophysiologic disruption. Early-onset preeclampsia poses a greater threat to maternal and fetal outcomes due to its association with severe placental insufficiency and heightened risk of pregnancy complications [33]. Management remains conservative, emphasizing close surveillance and often necessitating hospitalization [64]. Ultimately, delivery—via induction or cesarean section—remains the only definitive treatment, though it carries inherent risks for both mother and neonate [65].

### 5.1. Common Pathophysiological Pathways in Preeclampsia and Ferroptosis

Emerging evidence suggests that ferroptosis plays a significant role in pregnancy-related complications, particularly preeclampsia. Bioinformatics-based analyses have increasingly been employed to identify ferroptosis-related genes (FRGs) and characterize their differential expression in normal versus pathological pregnancies, with the goal of uncovering potential therapeutic targets. Clearly, the majority of studies investigating the role of ferroptosis in pregnancy complications focus primarily on preeclampsia and other hypertensive disorders.

In a study by He et al. [30], transcriptomic profiling of placental tissues from women with and without preeclampsia revealed several differentially expressed FRGs previously implicated in cardiovascular disease. These genes were found to participate as well, in pathways including ferroptosis and cysteine/methionine metabolism. Notably, *NDRG1*, *P4HA1*, and *LDHA* were significantly upregulated in quantification of preeclamptic placentas, while *IDO-1*—a regulator of vascular tone and inflammation—was the only FRG consistently downregulated [30]. These findings suggest a mechanistic role for *IDO-1* in the intersection of ferroptosis and preeclampsia pathophysiology. Additional studies have further substantiated this association. Placental tissues from preeclamptic pregnancies demonstrate increased expression of ferroptosis-associated markers—including *ALB*, *NOX4*, *CDKN2A*, *TXNRD1*, and *CAV1*—within the syncitiotrophoblast layer, reinforcing a potential pathogenic link [31].

Sulfiredoxin-1 (*SRXN1*), known for its antioxidant function, has also emerged as a candidate regulator of ferroptosis in preeclampsia. While typically associated with cancer pathways, *SRXN1* was found to be markedly downregulated in a murine model of preeclampsia, correlating with impaired trophoblast viability, invasion, and myometrial migration—features mediated through ferroptotic mechanisms [32]. These findings further emphasize the need to differentiate early-onset from late-onset preeclampsia, given their distinct molecular underpinnings.

Studies on late-onset preeclampsia have revealed similarly compelling results. Ortega et al. [33] identified elevated levels of key ferroptosis mediators—*NOX1*, *NOX2*, *TfRC*, *ALOX5*, *ACSL4*, *GPX4*, and malondialdehyde (MDA)—in placental tissues from affected pregnancies. Increased ferric iron (Fe^3+^) accumulation was also observed, supporting ferroptosis as a contributory mechanism in disease development [33].

Further support comes from work by Qi et al. [34], who examined *SIRT6*, a NAD^+^-dependent deacetylase involved in genomic stability and redox regulation. Their study reported elevated *SIRT6* expression in preeclamptic placentas, alongside suppressed antioxidant levels (glutathione and superoxide dismutase) and increased MDA concentrations [34]. These changes were associated with upregulation of the Nrf2/HO-1 pathway, suggesting a role for *SIRT6* in modulating ferroptotic responses.

Iron export mechanisms have also been implicated. Notably, *FPN* (Ferroportin) expression was significantly reduced in placental tissues from pregnancies complicated by preeclampsia and spontaneous preterm birth (SPTB) [35]. Functional silencing of *FPN* increased cellular susceptibility to ferroptosis, particularly when combined with erastin, a known ferroptosis inducer. Decreased *FPN* expression was also detected in fetal membranes from SPTB cases, highlighting the importance of iron homeostasis in maintaining placental integrity. Disruption of ferroptosis-regulating pathways may therefore contribute broadly to the pathogenesis of adverse pregnancy outcomes [35].

### 5.2. Potential Therapeutic Targets in Preeclampsia

The significance of studies connecting ferroptosis with distinct biomarkers in the context of gestational pathologies has great research potential and clinical impact, as treatments may rise if largely tested.

The study by Beharier et al. [66] identified PLA2G6, a member of the phospholipase A2 family, as a novel regulator of ferroptosis in human placental trophoblasts. PLA2G6 was found to be highly expressed in placental tissue, where it hydrolyzes hydroperoxide-phosphatidylethanolamines (Hp-PEs)—lipid peroxides that are known mediators of ferroptotic cell death [66]. By degrading Hp-PEs, PLA2G6 appears to play a protective role in limiting ferroptosis-associated damage. In PLA2G6 knockout (PLA2G6^−^/^−^) mice, loss of this enzymatic activity led to a marked increase in ferroptotic signaling, particularly via RSL3-induced pathways [66]. Furthermore, concurrent silencing of glutathione peroxidase 4 (GPX4)—a well-established ferroptosis inhibitor—exacerbated cell death in both PLA2G6-deficient and wild-type models. These findings suggest a synergistic relationship between PLA2G6 and GPX4 in maintaining cell viability. Moreover, in vivo experiments using mouse-derived placental cells exposed to hypoxia-reoxygenation injury reproduced similar results, reinforcing the clinical relevance of PLA2G6 as a potential therapeutic target in placental disorders characterized by oxidative stress and trophoblast dysfunction [66].

Empagliflozin, a widely used sodium-glucose co-transporter 2 (SGLT2) inhibitor, has demonstrated notable efficacy in managing heart failure with preserved ejection fraction (HFpEF), largely attributed to its glycemic modulation, although its precise mechanism of action remains incompletely understood till present [67]. In a recent study by Jiahao Tong et al., a murine model of antibody-induced preeclampsia was established using agonistic autoantibodies targeting the angiotensin II type 1 receptor (AT1-AA), allowing investigation into renal tubular epithelial involvement in preeclampsia pathogenesis [68]. Compared to controls, mice exposed to AT1-AA exhibited significantly greater injury to renal proximal tubular epithelial cells. Mechanistically, the observed damage was attributed to ferroptosis, as evidenced by elevated iron accumulation, glutathione depletion, and increased phospholipid hydroperoxides (PLOOH) in preeclamptic renal tissues. Notably, treatment with empagliflozin provided protective effects from ferroptosis reversing damage of proximal renal epithelial cells [68]. These findings suggest that empagliflozin may hold translational potential as a therapeutic strategy for ferroptosis-driven renal dysfunction in preeclampsia, warranting further investigation in clinical settings involving pregnant populations.

Puerarin, a naturally occurring isoflavone extracted from the root of Pueraria lobata (kudzu)—a staple of traditional Chinese medicine [69]—has garnered increasing interest for its therapeutic potential in cardiovascular and metabolic disorders [70]. In a murine model of preeclampsia, puerarin administration attenuated placental injury and was found to activate key antiferroptotic pathways, including the upregulation of glutathione peroxidase 4 (GPX4) and the cystine/glutamate antiporter (SLC7A11) [70]. In trophoblastic cells, puerarin suppressed reactive oxygen species (ROS) levels and downregulated heme oxygenase-1 (HO-1) and cAMP-response element binding protein (CREB). Notably, HO-1 deficiency led to compensatory upregulation of GPX4 and SLC7A11, suggesting a mechanistic link between the HO-1/CREB axis and the regulation of ferroptosis [70]. These findings provide compelling evidence that puerarin not only mitigates placental oxidative injury but also modulates ferroptotic signaling, thereby offering a promising therapeutic avenue for the treatment of preeclampsia.

Quercetin, a naturally occurring flavanol, has demonstrated potential in promoting endothelial repair, a critical target in the pathophysiology of preeclampsia [71]. While its direct association with canonical ferroptosis markers remains unclear, a study by Meiting Shi et al. showed that low-dose quercetin, bind to epidermal growth factor receptor (EGFR) signaling, improved uteroplacental perfusion and enhanced endothelial function in murine models [71]. The therapeutic promise of quercetin lies in its potential as a preventive and adjunctive agent in the management of preeclampsia and given the safety profile and bioactivity of naturally derived compounds, quercetin may represent a compelling alternative to conventional pharmacotherapies, within the restrictive landscape of pregnancy-safe medications [71].

All trans–retinoic–acid (ATRA) was related with ferroptosis in gestational complications by the study of [6,14,15] et al. which administration of ATRA in trophoblastic cells halted ferroptosis causing resistance to this iron–driven cell type of death by enhancing expression of HMOX-1 [72].

Collectively, these findings underscore the therapeutic promise of targeting ferroptosis and its regulatory networks to improve outcomes in pregnancies complicated by cardiovascular risk. Despite growing preclinical evidence supporting this strategy, no pharmacologic agents specifically targeting ferroptosis have yet entered clinical trials involving pregnant individuals with gestational pathologies. As such, the current body of knowledge remains confined to preclinical and translational models, highlighting an urgent need for clinical investigations in this population.

## 6. Gestational Diabetes Mellitus Pathogenesis

Gestational diabetes mellitus (GDM), defined as glucose intolerance first identified during pregnancy, affects approximately 14.7% of pregnant individuals worldwide [73]. Obesity is a primary risk factor, largely due to its role in promoting chronic inflammation, oxidative stress, and insulin resistance—key pathophysiologic processes that precipitate GDM [5]. The condition carries significant postpartum risks initially for mothers who face an elevated risk of mortality, while offspring are more likely to be born with macrosomia and predisposed to developing cardiovascular disease later in life. Insulin resistance in GDM is driven in part by proinflammatory cytokines secreted by placental cells, which sustain a hyperglycemic intrauterine environment [74]. Compounding this effect, dysfunctional maternal pancreatic β-cells often fail to meet the increased metabolic demands of pregnancy, further impairing glucose regulation and promoting disease progression [5].

### Gestational Diabetes Mellitus Molecular Pathways and Ferroptosis

Gestational diabetes mellitus (GDM) frequently emerges in the context of preexisting insulin resistance or impaired glucose homeostasis. Disruption of insulin signaling—such as aberrant serine phosphorylation of insulin receptor substrates (IRS-2 or IRS-3)—impedes downstream signal transduction, ultimately inhibiting the translocation of glucose transporter (GLUT) proteins to the cell membrane and reducing cellular glucose uptake [36,75]. In parallel, hyperactivation of anabolic signaling cascades, particularly the mechanistic target of rapamycin (mTOR) pathway, has been implicated in the development of insulin resistance and has been mechanistically linked to both GDM and ferroptosis [37]. Mitochondrial dysfunction also contributes to this pathophysiologic process; specifically, the failure to adequately upregulate ATP production during pregnancy is frequently observed in individuals with GDM, underscoring a potential mitochondrial role in disease onset and progression [76,77].

Beyond insulin resistance, impaired glucose regulation constitutes a core element in the pathogenesis of gestational diabetes mellitus (GDM). Human placental lactogen (HPL), a hormone secreted predominantly by the syncitiotrophoblast, plays a central role in this process [78]. Under physiological conditions, HPL promotes maternal lipolysis, thereby increasing circulating free fatty acids to meet maternal energy demands [78]. Simultaneously, HPL modulates glucose metabolism to support transplacental glucose transfer, ensuring adequate fetal nourishment. Importantly, HPL also exerts a direct inhibitory effect on insulin secretion—an adaptation believed to facilitate glucose availability for the fetus during pregnancy [78]. In addition to HPL, other factors such as cortisol and adiponectin have been implicated in the development of GDM [79]. Reduced adiponectin levels, frequently observed in GDM, may compromise its anti-inflammatory and insulin-sensitizing functions, thereby exacerbating metabolic dysregulation in affected individuals [80].

Ferroptosis and gestational diabetes mellitus (GDM) converge at multiple molecular intersections, notably through the involvement of key regulatory genes including GPX4, SLC7A11, ACSL4, and LPCAT3 [38]. GPX4 and SLC7A11 are central to antioxidant defense, functioning in glutathione biosynthesis and contributing to the maintenance of iron homeostasis and redox equilibrium [81]. In contrast, ACSL4 and LPCAT3 facilitate the incorporation of polyunsaturated fatty acids (PUFAs) into membrane phospholipids, thereby generating substrates susceptible to lipid peroxidation [82]. These coordinated molecular pathways highlight a mechanistic link between ferroptotic signaling and the metabolic disturbances characteristic of GDM.

Epigenetic modifications—including DNA methylation, histone acetylation, and mRNA expression—play a pivotal role in regulating genes associated with ferroptosis, thereby contributing to pathological processes that exacerbate pregnancy-related complications [83]. Specifically, silencing of antioxidant genes via DNA methylation and histone acetylation–mediated upregulation of ferroptosis activators provide mechanistic insight into this dysregulation [83]. In the setting of pregnancy, persistent hyperglycemia promotes intracellular iron accumulation, enhancing oxidative stress and triggering ferroptotic cell death [84]. Central to this process is the upregulation of SIRT3, which activates the AMPK–mTOR signaling pathway and suppresses glutathione peroxidase 4 (GPX4), a key antioxidant enzyme critical for ferroptosis resistance [84].

The combined effects of hyperglycemia and dyslipidemia further intensify the detrimental impact of diabetes on pregnancy outcomes. Notably, increased expression of the glucose transporter GLUT1 has been observed in placental tissue from pregnancies complicated by gestational diabetes mellitus (GDM). Silencing of GLUT1 expression was associated with downregulation of ferroptosis-related markers, suggesting a functional role for GLUT1 in modulating ferroptotic pathways [85]. Mechanistically, GLUT1 overexpression induces AMPK phosphorylation and decreases phosphorylation of acetyl-CoA carboxylase (ACC), thereby promoting lipid synthesis and enhancing lipid peroxidation—a hallmark of ferroptosis [85]. This lipid metabolic shift underscores the synergistic contribution of GLUT1 to ferroptosis and placental dysfunction, with downstream effects including fetal growth restriction [85].

Additionally, mitochondrial dysfunction—characterized by excessive reactive oxygen species (ROS) production and cellular energy imbalance—further exacerbates ferroptotic signaling. The RNA-binding protein CPEB4 has emerged as a key mediator of this process, promoting ferroptosis in trophoblasts through the transcriptional activation of pro-ferroptotic genes. These findings implicate CPEB4 as a potential contributor to GDM pathogenesis and highlight its value as a target for further investigation [86].

## 7. NETosis: A Novel Cell Death Mechanism in Pregnancy Complications

NETosis is a distinct form of programmed cell death involving the release of neutrophil extracellular traps (NETs), which are web-like structures composed of neutrophilic DNA, histones, neutrophil elastase (NE), and myeloperoxidase (MPO) [87]. While initially characterized as a host defense mechanism, NETosis has been increasingly implicated in sterile inflammatory states, including pregnancy-related complications such as preeclampsia and spontaneous abortion [9].

Emerging evidence suggests a pathophysiological interplay between NETosis and ferroptosis, two mechanistically distinct but interrelated forms of cell death. Experimental models have demonstrated that NET formation exacerbates ferroptosis through the generation of reactive oxygen species (ROS) and lipid peroxidation. For instance, in a murine model of intestinal ischemia–reperfusion injury, simultaneous activation of NETosis and ferroptosis contributed to endothelial damage, while pharmacologic inhibition of NETosis mitigated ferroptotic cell death [88]. Similar interactions have been observed in pulmonary thrombosis, where surface-expressed phosphatidylserine on ferroptotic platelets promotes NET formation, triggering the ASK-1 inflammasome pathway and driving vascular injury [39]. In abdominal aortic aneurysm, NET-induced destabilization of the mitochondrial carrier SLC25A11 depletes mitochondrial glutathione, initiating ferroptosis in vascular smooth muscle cells. These mechanistic insights highlight a shared inflammatory axis with therapeutic potential [89].

In pregnancy, NETosis is increasingly recognized as a contributor to maternal and fetal morbidity [90]. Both suicidal and non-suicidal (vital) forms of NETosis have been implicated in gestational pathologies marked by endothelial dysfunction [9]. Neutrophils isolated from pregnancies complicated by preeclampsia exhibit delayed apoptotic marker expression and heightened NET formation [90]. Elevated levels of adhesion molecules—such as L-selectin on CD15+ neutrophils—promote leukocyte-endothelial interactions [40], while increased expression of intercellular adhesion molecule-1 (ICAM-1) and interleukin-8 (IL-8) at the placental interface further recruits neutrophils, amplifying inflammation, oxidative stress, and hypoxia [41].

Quantifiable biomarkers of NETosis, including citrullinated histones, neutrophil elastase (NE), and MPO, are elevated in preeclampsia and correlate with disease severity [42]. A study by de Buhr et al. [91] reported significantly increased circulating NET components in women with preeclampsia compared to controls, implicating NETosis in disease progression. In turn, excessive NET accumulation at the maternal–fetal interface induces trophoblast dysfunction and contributes to placental insufficiency [92].

High-mobility group box 1 (HMGB1), a damage-associated molecular pattern released during NETosis, has emerged as a central mediator of trophoblast injury. In preeclampsia, HMGB1 promotes inflammation and oxidative stress at the placental interface. Its inhibition reduces cytokine production and improves trophoblast viability, identifying HMGB1 as a candidate therapeutic target. Although causal molecular mechanisms of spontaneous abortion are not discussed in this article, it is worth mentioning that confirmed HMGB1 upregulation—particularly in response to lipopolysaccharide-induced inflammation—induces ferroptosis through stabilization of ACSL4, linking NETosis-associated inflammation with trophoblast loss. In GDM, hyperglycemia induces HMGB1 release from placental trophoblasts, which activates Toll-like receptor 4 (TLR4) signaling, leading to increased production of inflammatory cytokines (including IL-8), impaired trophoblast migration, and a proinflammatory placental environment [93]. Moreover, pharmacologic blockade of HMGB1 or ACSL4 significantly attenuates this cell death pathway, underscoring the relevance of the HMGB1/ACSL4 axis in early pregnancy loss [43,94].

Recognizing these convergent inflammatory mechanisms has stimulated interest in repurposing established agents. Aspirin and metformin, widely used for cardiovascular and metabolic indications, are currently under investigation for their potential to mitigate NETosis and related endothelial injury in preeclampsia [95,96]. Ongoing clinical trials will be essential to determine their efficacy and safety of those agents in this particular context of gestation pathologies.

## 8. Endothelial Pathophysiology in Pregnancy: Mechanistic Insights from HUVEC Models of Preeclampsia and Hyperglycemia

Human umbilical vein endothelial cells (HUVECs) have emerged as a valuable model for investigating pregnancy-related vascular complications, particularly their involvement in ferroptosis-and NETosis-mediated pathophysiological processes [97]. Due to their well-characterized response to oxidative and inflammatory stressors, HUVECs are frequently employed in vitro to elucidate mechanisms underlying endothelial dysfunction, especially in the context of preeclampsia. However, the literature remains disproportionately focused on in vitro paradigms, with limited translation to in vivo systems. In vivo observed phenomena, such as shear stress forces, remain understudied due to the inherent limitations in the design and availability of appropriate in vitro models—despite their established relevance to endothelial pathophysiology in preeclampsia [98]. Emerging data support a mechanistic link between maternal endothelial dysfunction and ferroptosis triggered by placental hypoxia. Specifically, lipid peroxides secreted from extracellular vesicles of damaged endothelial cells have been shown to impair angiogenic function in HUVECs [99]. Restoration of iron homeostasis can reverse endothelial damage, further implicating ferroptosis as a key driver of vascular impairment in gestational disorders such as preeclampsia. Although direct investigations of NET formation in HUVECs during pregnancy are scarce, existing evidence highlights the interplay between oxidative stress, ferroptosis, and endothelial activation—processes known to promote NETosis and exacerbate cardiovascular complications in pregnancy [99]. In addition to preeclampsia, HUVECs have been implicated in the pathogenesis of gestational hyperglycemia. Zheng et al. demonstrated that HUVECs isolated from hyperglycemic pregnancies exhibit elevated expression of senescence-associated markers, including SA-β-gal, p16, p21, and p53 [100]. De novo exposure of naïve HUVECs to hyperglycemic conditions replicated these findings and further induced markers of endothelial dysfunction such as von Willebrand factor (vWF), CCL2, ICAM-1, and the anti-apoptotic protein BCL2. These results confirm that sustained hyperglycemia fosters endothelial senescence, impairs angiogenesis, and promotes chronic inflammation—hallmarks of vascular pathology in pregnancy. Complementary transcriptomic analyses have revealed altered gene expression profiles in HUVECs derived from women with gestational diabetes mellitus (GDM), particularly in pathways associated with metabolism, inflammation, oxidative stress, and insulin signaling [101]. Notably, endothelial injury is not limited to hyperglycemia alone; abrupt transitions to hypoglycemic states also precipitate oxidative stress via reactive oxygen species (ROS) accumulation and activation of the TGF-β signaling pathway. This cascade induces connective tissue growth factor (CTGF) expression, thereby modulating the extracellular matrix and influencing local cellular architecture [101].

## 9. Clinical Implications and Future Directions

Advancements in our understanding of ferroptosis and NETosis have the potential to reshape the clinical approach to cardiovascular complications arising during pregnancy. By identifying these unique forms of regulated cell death and immune activation as contributors to conditions such as preeclampsia, gestational hypertension, and peripartum cardiomyopathy, a new perspective emerges on the underlying mechanisms of these disorders. This insight serves to a more refined risk assessment, as molecular markers associated with lipid peroxidation or neutrophil extracellular trap formation could, in the future, be incorporated into screening protocols to help identify women at heightened risk for adverse cardiovascular outcomes both during and after pregnancy. Such biomarkers may enable clinicians to initiate closer surveillance and timely interventions, ultimately improving maternal and fetal prognosis.

Therapeutically, targeting the molecular pathways underlying ferroptosis and NETosis presents a promising approach for attenuating tissue injury and inflammation associated with pregnancy-related cardiovascular disorders. Strategies aimed at modulating iron homeostasis, augmenting endogenous antioxidant defenses, or suppressing excessive neutrophil activation have been investigated in other clinical settings and, following thorough safety and efficacy evaluations, may be adapted for use in pregnant populations. The development of pharmacological agents that inhibit iron-driven lipid peroxidation or prevent the aberrant release of neutrophil extracellular traps holds potential to complement existing therapeutic regimens for these multifactorial conditions.

Despite these promising avenues, translating basic scientific discoveries into clinical practice remains challenging. There is a need for comprehensive research involving large and diverse cohorts of pregnant women to validate the clinical utility of these molecular pathways as diagnostic or prognostic tools. Additionally, the unique physiological changes in pregnancy must be considered, as they may influence both the activity and the consequences of ferroptosis and NETosis. Future investigations should aim to clarify how these processes unfold in maternal and placental tissues over the course of gestation, and to assess their long-term effects on both maternal and offspring health.

## 10. Limitations

This review is narrative in nature and therefore may be vulnerable to search and reference biases, as the evidence summarized was not identified through a pre-registered, comprehensive systematic search or assessed with meta-analytic methods. While the synthesis integrates current knowledge on ferroptosis and NETosis in pregnancy complications, it does not quantitatively estimate effect sizes or directly compare outcomes across studies. Moreover, heterogeneity in experimental models, study designs, and patient populations limits the generalizability of some findings. We acknowledge that ongoing research and emerging data may further refine these interpretations. Future systematic reviews and meta-analyses—including formal risk-of-bias assessments—are warranted to more rigorously evaluate the evidence base, mitigate publication and citation biases, and support clinical translation.

## 11. Conclusions

In summary, the exploration of ferroptosis and NETosis in the context of pregnancy-associated cardiovascular complications represents a rapidly progressing field with significant clinical relevance. Ongoing research is essential to clarify their mechanistic roles, discover reliable biomarkers, and develop interventions that are both safe and effective for pregnant women. A deeper understanding of these pathways holds promise for advancing risk prediction, prevention, and management strategies, ultimately reducing the burden of cardiovascular disease in mothers and their children.

## Abbreviations

The following abbreviations are frequently used in this manuscript:

ACSL	Acyl-CoA Synthetase Long-chain
DMT	Divalent Metal Transporter
GDM	Gestational Diabetes Mellitus
GPX4	Glutathione Peroxidase 4
GSH	Glutathione
LPCAT	Lys phosphatidylcholine Acyltransferase
NETs	Neutrophil Extracellular Traps
NLRP	NOD-LRR-and Pyrin Domain-Containing Protein
PE	Preeclampsia
PLOOH	Phospholipid Hydroperoxides
PUFAs	Polyunsaturated Fatty Acids
ROS	Reactive Oxygen Species
Tf	Transferrin
TfR	Transferrin Receptor
